# Functional analysis of AP2/ERF family members in flavonoid biosynthesis of wolfberry

**DOI:** 10.3389/fpls.2025.1632482

**Published:** 2025-09-02

**Authors:** Ronghui Li, Shijia Xia, Qiyuan Long, Cheng Jin, Haoxia Li, Yuhui Xu, Yuchao Chen, Xiaoyan Gan, Yuanyuan Zhang, Jianhua Zhao

**Affiliations:** ^1^ School of Breeding and Multiplication (Sanya Institute of Breeding and Multiplication), Hainan University, Sanya, China; ^2^ School of Tropical Agriculture and Forestry, Hainan University, Haikou, Hainan, China; ^3^ College of Biological Science and Engineering, North Minzu University, Yinchuan, China; ^4^ National Wolfberry Engineering Research Center, Wolfberry Science Research Institute, Ningxia Academy of Agriculture and Forestry Sciences, Yinchuan, China; ^5^ Agricultural Biotechnology Centre, Ningxia Academy of Agriculture and Forestry Sciences, Yinchuan, China

**Keywords:** AP2/ERF family, wolfberry, flavonoids, LbAP2/ERF089, transcription factor

## Abstract

Flavonoids, a group of major bioactive ingredients, contribute to the nutritional and medicinal properties of wolfberry (*Lycium barbarum*). *APETALA2/Ethylene response factors* (*AP2/ERFs*) are widely distributed in plants and play crucial roles in regulating growth, development, and stress responses. However, the knowledge of *AP2/ERF* genes in wolfberry remains limited, and specific *AP2/ERF* family members involved in flavonoid biosynthesis have not been identified. Here, we systematically identified and characterized AP2/ERF proteins in wolfberry and identified key *AP2/ERF* family members involved in regulating *flavonoid biosynthesis. LbAP2/ERF* genes were identified via BLASTP and HMM analysis, using Arabidopsis AP2/ERF as queries and the *L. barbarum* genome. Gene duplication was analyzed with DupGen_finder, fruit RNA-seq was tested to determine expression profiles across five developmental stages, and *LbAP2/ERF089* function was verified using transient overexpression and dual-luciferase assays. A total of 148 genes belonging to the *L. barbarum AP2/ERF* (*LbAP2/ERF*) family were identified, with dispersed duplication likely being the primary driver of their amplification. The *LbAP2/ERF* genes exhibit distinct expression profiles during different stages of fruit development, indicating their potential importance in wolfberry fruit development. Based on the metabolite and gene network analysis, a series of *LbAP2/ERF* genes involved in flavonoid biosynthesis were identified, including *LbAP2/ERF089, LbAP2/ERF011, LbAP2/ERF068*, and *LbAP2/ERF099*. Functional analysis further revealed that *LbAP2/ERF089* positively regulates flavonoid synthesis by activating the expression of biosynthetic genes *LbLAR* and *LbDFR*. Overall, our results provide new insights into the transcriptional regulation of flavonoid and anthocyanin biosynthesis and offer valuable genetic resources for enhancing the nutritional and medicinal value of wolfberry.

## 1 Introduction

Wolfberry, belonging to the genus *Lycium* within the Solanaceae family, plays a pivotal role as a valuable medicinal and edible resource in China. It is mainly distributed in the northwestern regions of China and certain areas in the Mediterranean Basin. The cultivation and trade of wolfberry fruits have generated substantial economic benefits for these regions (Amagase and Farnsworth, 2011; Jin et al., 2013). Research has demonstrated that wolfberry fruits are abundant in flavonoids such as rutin, quercetin, kaempferol, and myricetin (Miean and Mohamed, 2001; Bondia-Pons et al., 2014). These compounds possess a wide range of beneficial biological activities, including antioxidant, anticancer, anti-inflammatory, antibacterial, antiviral, anti-tumor, and neuroprotective effects (Amagase et al., 2009a, 2009; Jiang, 2014). Thus, the significant accumulation of flavonoids is often considered a defining characteristic of high-quality wolfberry fruits (Wang et al., 2018).

Flavonoids, including anthocyanins, are biosynthesized through the phenylpropanoid pathway (Sainz et al., 1997; Winkel-Shirley, 2001; Zhong et al., 2020). Phenylpropanoid metabolism initiates with the deamination of phenylalanine catalyzed by phenylalanine ammonia-lyase (PAL), generating *trans*-cinnamic acid. This intermediate is subsequently converted by cinnamate 4-hydroxylase (C4H) and 4-coumarate coenzyme A ligase (4CL) to form *p*-coumaroyl-CoA. *p*-Coumaroyl-CoA condenses with three malonyl-CoA molecules catalyzed by chalcone synthase (CHS) and chalcone isomerase (CHI), followed by subsequent reduction and hydroxylation reactions to generate various flavonoids (Liu et al., 2019a; Shan et al., 2020). Most enzymes in flavonoid biosynthesis are encoded by a single gene in Arabidopsis, while in poplar, grape, and apple, these enzymes are encoded by multiple genes, though their functions remain mostly conserved (Sparvoli et al., 1994; Kim et al., 2003; Tsai et al., 2006). Similarly, the identified structural genes *PAL*, *flavonoid 3′-hydroxylase* (*F3′H*), *leucoanthocyanidin reductase* (*LAR*), and *anthocyanidin reductase* (*ANR*) in wolfberry that encode enzymes involved in flavonoid biosynthesis demonstrate functional conservation within the pathway (Zeng et al., 2014). As well as structural genes, various transcription factors such as MYB, bHLH, WD40, WRKY, and AP2/ERF are involved in the regulation of plant flavonoid biosynthesis (Zhao et al., 2021a; Gao et al., 2022; Wang et al., 2022). The transcription factors MYB11, MYB12, and MYB111, which belong to subgroup 7 of the R2R3-MYB family, have been reported to regulate flavonol biosynthesis (Stracke et al., 2007, 2009). *VvWRKY26* regulates the hydroxylation steps of flavonoids in *Vitis vinifera* (Amato et al., 2017). The *MdAP2–34* stimulates flavonoid accumulation via the flavonoid biosynthetic pathway by directly binding to and activating the *MdF3′H* promoter activity in *Malus domestica* (Han et al., 2022). In *Lycium*, *LrMYB1*, *LrAN2*, *LrMYB113*, and *LrAN11* were shown to be potential regulators of flavonoid biosynthesis (Ye et al., 2021; Wang et al., 2020). Recently, *LrMYB94* and *LrWRKY32* were shown to collaboratively regulate the accumulation of quercetin-3-O-rutinoside and quercetin-3-O-rhamnoside in *L. ruthencum* by upregulating the expression of *LrFLS*, *LrCHS*, *LrF3H*, and *LrCYP75B1* (Du et al., 2024).

The AP2/ERF transcription factor family is plant-specific and constitutes one of the largest transcription factor families in plants, with typically over 100 members in each species (Gao et al., 2020). Based on the number and type of conserved domains present, the AP2/ERF transcription factors can be divided into five subfamilies, including AP2 (APETALA2), RAV (Related to ABI3/VP1), DREB (Dehydration responsive element binding), ERF (Ethylene-responsive factor), and Soloist (Mizoi et al., 2012). The DREB and ERF subfamilies can be further subcategorized into six groups: clade B1-B6 and clade A1-A6 (Riechmann and Meyerowitz, 1998). These genes regulate various aspects of plant secondary metabolism, development, fruit ripening, and defense responses (Gao et al., 2020). For example, CitERF32, CitRAV1, and CitERF33 form a DREB-RAV transcription complex that enhances the accumulation of flavones and flavonols in citrus by interacting with the CitCHIL1 protein (Zhao et al., 2021a). The expression of *LrAP2/ERF16* is associated with the accumulation of anthocyanins in *Lycoris* flower petals (Wang et al., 2023). In *Solanum lycopersicum*, the overexpression of *SlERF.G3* activates the expression of *SlFLS* and early genes in the flavonoid biosynthetic pathway, leading to increased flavonol accumulation in the fruit (Li et al., 2020b). Recent studies have shown that *LbERF5.1* modulates carotenoid accumulation by interacting with *LbCCD4.1* in *Lycium* (Zhao et al., 2023). However, the systematic identification and study of *AP2/ERF* genes in wolfberries are still lacking, and their involvement in regulating flavonoid biosynthesis in wolfberry fruit remains unclear.

In this study, we systematically identified and characterized 148 AP2/ERF proteins in wolfberry through phylogeny, gene structure, conserved domains, and gene duplication events. A series of potential genes involved in the flavonoid biosynthetic pathway were identified based on metabolite and gene network analyses. Further functional analysis revealed the role of a key *AP2/ERF* gene, *LbAP2/ERF089*, in regulating flavonoid and anthocyanin biosynthesis.

## 2 Materials and methods

### 2.1 Plant materials and treatment

The Ningqi No. 1 (NQ) and Ningxia Huangguo (NX) were grown at the National Wolfberry Germplasm Resource Nursery of the Ningxia Academy of Agricultural and Forestry Sciences (38°38′N, 106°9′E, altitude 1100 meters). All the trees were 8 years old, planted with a row spacing of 2 meters and an individual spacing of 1 meter. During the peak fruiting period from June to July 2021, we sampled the fruits from three asexual plants of each variety at five developmental stages (S1-S5). After collecting the samples, all materials were frozen in liquid nitrogen and stored at -80 °C for RNA and metabolite extraction. Hydroponic seedlings of NQ were used for transient overexpression experiments.

### 2.2 Identification of *AP2/ERF* genes in wolfberry

The sequences of *Arabidopsis thaliana* AP2/ERF (AtAP2/ERF) proteins, retrieved from the Arabidopsis Information Resource (https://www.arabidopsis.org/), were used as queries for BLASTP searches to identify AP2/ERF family proteins, with the search restricted to entries having E-values < 1×10^-10. The genome sequence of *L. barbarum* was downloaded from the NCBI database (https://www.ncbi.nlm.nih.gov/) with accession number PRJNA640228 (Cao et al., 2021). The genome sequences of *S. lycopersicum, P. inflata, Nicotiana tabacum, Capsicum annuum, Solanum melongena*, and *Solanum tuberosum* were downloaded from the Solanaceae Genomics Network (https://solgenomics.net/). The genome sequences of *Chromochloris zofingiensis*, *Chlamydomonas reinhardtii*, *Marchantia polymorpha*, *Physcomitrium patens*, *Ginkgo biloba*, *Zea mays*, *Sorghum bicolor*, *Hordeum vulgare*, *Oryza sativa*, *A. thaliana*, *Gossypium hirsutum*, *Phaseolus vulgaris*, *Cucumis melo*, *Carya illinoensis*, *Prunus persica*, *M. domestica*, *V. vinifera*, *Actinidia chinensis*, and *Coffea arabica* were downloaded from the NCBI database (https://www.ncbi.nlm.nih.gov/). Concurrently, the AP2 domain (PF00847) from Pfam (http://pfam.xfam.org/) was utilized to construct a Hidden Markov Model (HMM), which was applied to screen protein datasets using HMMER3.0 (Wheeler and Eddy, 2013) under the same statistical threshold. Conserved AP2 domain validation was conducted across multiple databases, including SMART (http://smart.embl-heidelberg.de/), CDD (https://www.ncbi.nlm.nih.gov/Structure/cdd/cdd.shtml), and Pfam database (http://pfam.xfam.org/) to confirm their presence in candidate sequences. Protein sequences lacking the AP2 domain and redundant sequences were manually removed. Based on the deduced amino acid sequences of all AP2/ERF proteins, the ProtParam tool (https://web.expasy.org/protparam/) was used to predict their isoelectric point (pI) and molecular weight (MW). Subsequently, the online platforms ExPASy (https://web.expasy.org/protparam/) and WoLF PSORT (https://wolfpsort.hgc.jp/) were used to predict the physicochemical properties and subcellular localization of the gene family, respectively.

### 2.3 Gene structure and conserved motif analysis

Conserved motif analysis of LbAP2/ERFs was performed using the MEME suite (https://meme-suite.org/meme/tools/meme), with parameters set as a maximum of 10 motifs and a minimum motif width of 6. A maximum likelihood (ML) phylogenetic tree was constructed using IQ-TREE (Minh et al., 2020), with the optimal substitution model VT+R6 selected via ModelFinder. Subsequently, TBtools (Chen et al., 2020) was employed to co-visualize gene structures, phylogenetic relationships, and conserved motif distributions, enabling integrated comparative analysis.

### 2.4 Gene duplication and evolutionary analysis

The full protein sequences of AP2/ERF from Arabidopsis and wolfberry were used for the phylogenetic tree construction. Multiple sequence alignment was performed using the ClustalW algorithm implemented in MEGA-X (Kumar et al., 2018), followed by ML tree inference with 1000 bootstrap replicates. OrthoFinder (Emms and Kelly, 2019) was employed to construct evolutionary relationships across 26 species, including *C. zofingiensis*, *C. reinhardtii*, *M. polymorpha*, *P. patens*, *G. biloba*, *Z. mays*, *S. bicolor*, *H. vulgare*, *O. sativa*, *A. thaliana*, *G. hirsutum*, *P. vulgaris*, *C. melo*, *C. illinoensis*, *P. persica*, *M. domestica*, *V. vinifera*, *A. chinensis*, *C. arabica*, *P. inflata*, *N. tabacum*, *L. barbarum*, *C. annuum*, *S. melongena*, *S. tuberosum*, and *S. lycopersicum*. Resulting trees were visualized using the ggplot2 package (Ito and Murphy, 2013) for visualization in the R statistical environment.

Inter-species and intra-species collinearity analysis was conducted with MCScanX (Wang et al., 2012), while Solanaceae-specific AP2/ERF phylogenetic trees were reconstructed using IQ-TREE2 (Minh et al., 2020) and visualized via iTOL (https://itol.embl.de/). Synonymous (Ks) and nonsynonymous (Ka) substitution rates for syntenic gene pairs were calculated using KaKs_calculator2.0 (Wang et al., 2010). Paralogous gene pairs derived from whole-genome duplication (WGD), tandem duplication (TD), proximal duplication (PD), transposed duplication (TRD), and dispersed duplication (DSD) were identified using the DupGen_finder pipeline (Qiao et al., 2019), with stringent filtering based on duplication event criteria.

### 2.5 *LbAP2/ERF* genes expression analysis

RNA-Seq data were obtained from previously published studies (Zhao et al., 2023), which included transcriptome profiles of both NQ and NX fruits at five developmental stages (S1-S5). The adapter sequences, low-quality reads (quality score < 15), and poly (A/T) tails were removed from raw reads using fastp (Chen et al., 2018) with default parameters except for setting the minimum length of reads to be retained as 30. The Hisat2 (Kim et al., 2015) software aligned clean reads to the reference genome using the default splice-junction database provided by the software and other default parameters. FeatureCounts software (Liao et al., 2014) estimated transcript abundances considering gene lengths and using the default counting mode. The Transcripts Per Million (TPM) measured the expression levels of the *AP2/ERF* genes. The expression patterns of each *AP2/ERF* gene in NQ and NX fruits across developmental stages were visualized as a heatmap using R software (https://www.r-project.org/).

### 2.6 Correlation and expression network analysis and WGCNA

Weighted Gene Co-expression Network Analysis (WGCNA) (Langfelder and Horvath, 2008) was applied to construct the co-expression network, using RNA-seq data from both NQ and NX fruits at five developmental stages (S1-S5) (Zhao et al., 2023). The co-expression association of all genes was extracted from RNA-Seq data in a non-targeted way, and the highly interconnected modules of co-expression genes were detected. Using the Hmisc package in R (Yadav and Roychoudhury, 2018), we calculated the correlation between the expression levels of *AP2/ERF*, structural genes involved in regulating flavonoid biosynthesis, and the content of flavonoid metabolites. We computed the Pearson correlation coefficients and significance *p*-values for these relationships. Utilize the pheatmap package in R (https://cran.r-project.org/web/packages/pheatmap/index.html) to generate a heatmap. Visualizing the interaction networks using Cytoscape v3.10.0 (Shannon et al., 2003).

### 2.7 Flavonoid metabolites analysis

Ultra Performance Liquid Chromatography-Tandem Mass (UPLC-MS/MS)-Based Quantitative metabolomics and targeted metabolomics methods (Novogene, China) were used to construct metabolomics libraries from samples of NQ and NX. The detailed procedure for the detection and analysis of metabolites was described before (Zhao et al., 2024).

### 2.8 Overexpression *LbAP2/ERF089* in wolfberry leaves

Total RNA was isolated from NQ leaves using a Takara RNA extraction kit, and single-stranded cDNA of *LbAP2/ERF089* was synthesized with the Reverse Aid First-strand cDNA Synthesis Kit (Thermo Fisher Scientific). Based on wolfberry genome and transcriptome data, primers (**Supplementary Table S1**) were designed to amplify the CDS of *LbAP2/ERF089* by Primer3 (Untergasser et al., 2012). PCR conditions were: pre-denaturation at 98°C for 3 min, followed by 30 cycles of 98°C denaturation for 10 s, 58°C annealing for 30 s, 68°C extension for 1 min, and a final extension at 68°C for 5 min. Purified amplicons were cloned into the expression vector pCambia1300-35S-GFP for Sanger sequencing. Transient overexpression in the wolfberry leaves was conducted following the protocols as described (Zhao et al., 2023). One-month-old hydroponically grown NQ seedlings were employed for transient overexpression assays. NQ seeds were surface-sterilized by soaking in pure water for ten minutes to remove impurities, followed by rinsing with 75% ethanol for 40 seconds, treating with 5% sodium hypochlorite for fifteen minutes, and finally washing with sterile water three times. The treated seeds were inoculated onto 1/2MS medium and cultured at 25°C under a 14h light/10h dark cycle with a light intensity of 4000 lx until the formation of two cotyledons. The seedlings were then transplanted into hydroponic tanks containing MS solution; the tanks were initially covered with plastic wrap (which was removed when the leaves touched it), and the seedlings were cultured until they reached one month of age. The pCambia1300-35S-*LbAP2/ERF089*-GFP vector and the empty vector were respectively transformed into Agrobacterium tumefaciens strain GV3101; the transformed Agrobacteria were subsequently suspended in an infection buffer consisting of 5 g/L D-glucose, 50 mM MES (pH 5.6), 2 mM Na_3_PO_4_·12_2_O, and 0.1 mM acetosyringone. The bacterial suspension was adjusted to an OD_600_ of 1.0 and incubated at 20-25°C for 1–2 hours. A 1 mL syringe without a needle was used to inject the bacterial suspension into the abaxial surface of the 3^rd^-5^th^ true leaves until a moist area appeared (**Supplementary Figure S1**). After infiltration, the plants were first cultured at 25°C in the dark for 12 hours, and then cultured at 25°C under a 14h light/10h dark cycle with a light intensity of 4000 lx for 72 hours. This generated *LbAP2/ERF089*-overexpressing plants, with pCambia1300-35S-GFP serving as the control. Samples were harvested for qRT-PCR and metabolome analyses, with at least 15 leaves selected for each biological replicate and a total of 3 replicates performed. Subsequently, the expression of *LbAP2/ERF089* in overexpression plants was determined by quantitative real-time PCR (qRT-PCR). The qRT-PCR primers used in this study were designed using Primer3 (Untergasser et al., 2012) (**Supplementary Table S1**). Using BIO-RAD CFX Connect™ Real-Time PCR System with *L. barbarum Elongation factor 1 alpha* (*LbEF1a*) as internal reference, qRT-PCR was performed according to our previous report (Zhao et al., 2021b), employing the 2^-ΔΔCT^ method to calculate gene expression levels (Livak and Schmittgen, 2001). Additionally, metabolites in NQ leaves overexpressing *LbAP2/ERF089* were quantified using UPLC-MS/MS-based metabolomics.

### 2.9 Transient transcription dual-luciferase assay

The 1000-bp upstream sequences of *LbLAR* and *LbDFR* were regarded as potential promoters. Genomic DNA from NQ leaves was extracted via the cetyltrimethylammonium bromide method. Primers (**Supplementary Table S1**) for amplifying these promoter sequences were designed using Primer3 (Untergasser et al., 2012), with PCR cloning conditions similar to those for *LbAP2/ERF089*. Promoters of *LbLAR* and *LbDFR* were amplified and cloned into the modified pH2GW7 vector (PJG094) containing the firefly luciferase (fLUC) gene and the Renilla luciferase gene (rLUC) as reporters, while the *LbAP2/ERF089* cDNA was cloned into the PJF754 (pEAQ-HT-DEST2) vector as an effector. The plasmids were transferred into *Agrobacterium tumefaciens* EHA105 by electroporation and co-infiltrated into *Nicotiana benthamiana* leaves. The luciferase activities were measured using the Dual-Luciferase Reporter Assay System (Promega) according to the manufacturer’s instructions. The relative reporter gene expression levels were expressed as the ratio of firefly LUC to Renilla luciferase (LUC/REN). Three independent transformations for each sample were performed.

### 2.10 Statistical analyses

Statistical analyses were conducted using GraphPad Prism 8 software. Student’s *t*-test was used for pairwise comparisons (*, *p* < 0.05; **, *p* < 0.01; ***, *p* < 0.001).

## 3 Results

### 3.1 Identification and phylogenetic analysis of *LbAP2/ERF* genes in wolfberry

**Figure 1:**
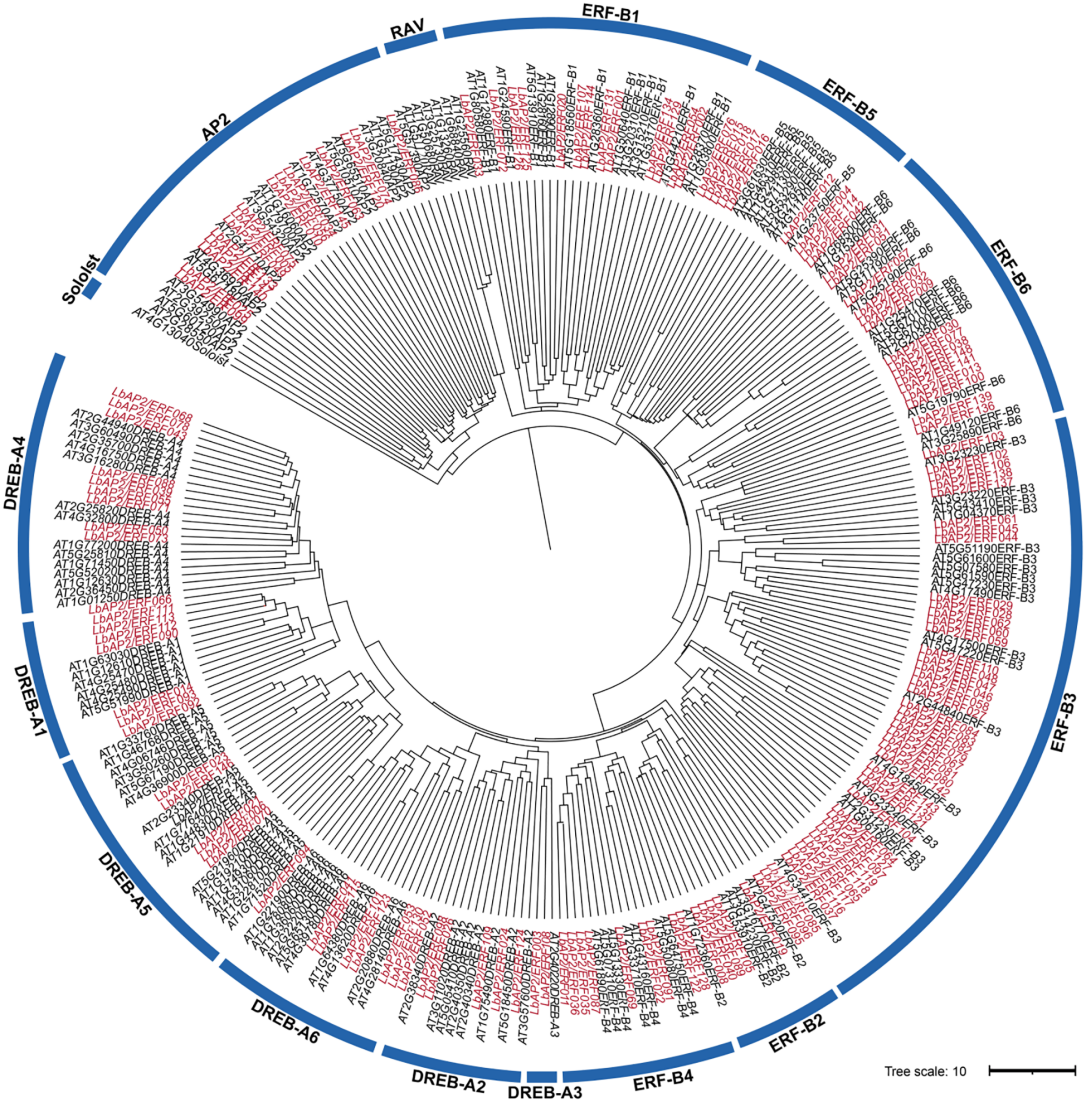
Phylogenetic analysis among AP2/ERF proteins of *L. barbarum* (NQ) and *A. thaliana.* The genes in *L. barbarum* are marked in red, while those in *A. thaliana* are marked in black.

A total of 148 high-confidence members of the *AP2/ERF* gene family were identified in the genome of *L. barbarum* through comprehensive analysis utilizing BLASTP and HMM. The *LbAP2/ERF g*enes are distributed across all 12 chromosomes of wolfberry and are named according to their chromosomal positions, ranging from *LbAP2/ERF001* to *LbAP2/ERF148.* These members display a wide range of amino acid lengths, varying from 126 to 1435 residues (**Supplementary Tables S2**, **S3**). To explore the phylogeny of *LbAP2/ERF* genes, a ML phylogenetic tree was constructed, including 148 LbAP2/ERF and 146 AtAP2/ERF proteins (**Figure 1**). Analysis showed LbAP2/ERFs, excluding RAV and Soloist, are distributed across AP2, ERF (B1-B6), and DREB (A1-A6) subfamilies, with ERF and DREB clearly categorized.

### 3.2 Duplication and evolutionary analysis of the *AP2/ERF* gene family

**Figure 2:**
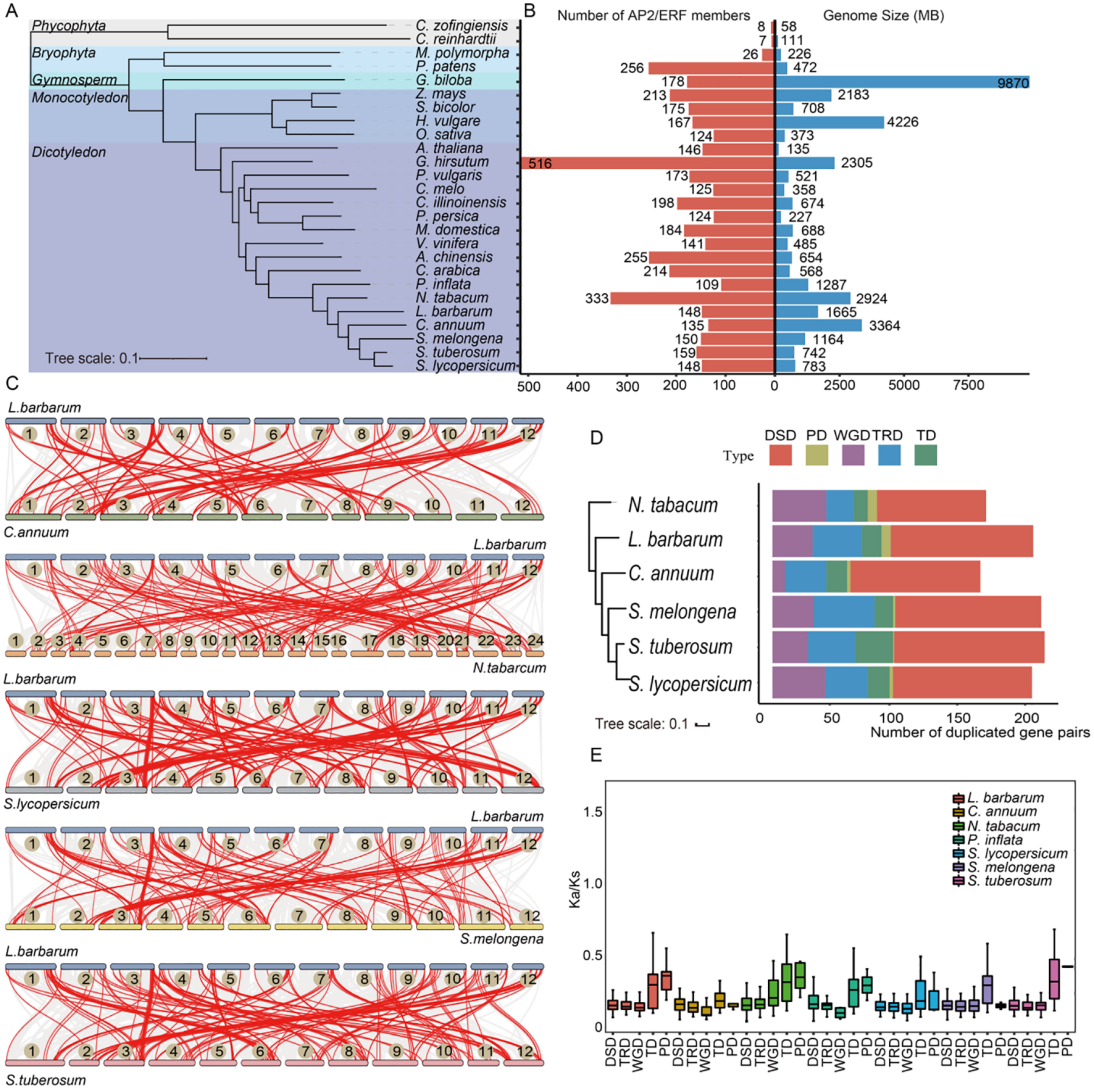
Evolutionary and gene duplication events analysis in multiple plants. **(A)** The species tree of the AP2/ERF family was constructed based on OrthoFinder. All selected plants were divided into five subgroups, distinguished by different colored branches. **(B)** The statistics of genome size and number of *AP2/ERF* family members for each species. **(C)** Synteny analyses of *LbAP2/ERF* genes between *L. barbarum* (NQ) and five other Solanaceae species. The gray lines in the background indicate the collinear block with wolfberry and other five plant species genomes, while red lines highlight syntenic *AP2/ERF* gene pairs. **(D)** The number of different modes of duplicated gene pairs in each species. The x-axis represents the number of duplicated gene pairs. The y-axis represents the species tree of Solanaceae species. **(E)** Ka/Ks ratios of Solanaceae species.

To assess the evolutionary status of the *AP2/ERF* gene family in major plant lineages, a total of 4,650 AP2/ERF proteins identified in 26 plant species were used to construct a species tree. OrthoFinder showed that *AP2/ERF* genes were present in all selected species (**Figures 2A, B**). Two species of phycophyta, *C. zofingiensis* and *C. reinhardtii*, contain only eight and seven AP2/ERF proteins respectively. In contrast, seed plants have a much higher number of AP2/ERF proteins, with their counts far exceeding those in algal species. Among seed plants, monocotyledonous species such as *O. sativa* (124 proteins) and *Z. mays* (213 proteins, the highest number) displayed substantial variation. Dicotyledonous and monocotyledonous plants were distinctly clustered into different branches on the species tree. Notably, the number of *AP2/ERF* genes was not strongly correlated with species genome size, suggesting potentially distinct evolutionary processes for *AP2/ERF* genes across different species.

To further determine the evolutionary trajectory of the *AP2/ERF* gene family in Solanaceae species, an analysis of the evolution and duplication events of these genes was performed. In the *L. barbarum* genome, we identified 59 non-redundant *AP2/ERF* genes as paralogous genes, forming 35 paralogous gene pairs distributed across its 12 chromosomes, with no clustering observed in specific chromosomal regions (**Supplementary Figure S2**). *L. barbarum* and five other Solanaceae species genomes were compared to investigate the gene collinear relationship in Solanaceae species (**Figure 2C**, **Supplementary Table S4**). The results reveal that the genome of *L. barbarum* shares collinear relationships with that of *C. annuum*, *N. tabacum*, *S. lycopersicum*, *S. melongena*, and *S. tuberosum*, accounting for 119, 154, 180, 130, and 170 *AP2/ERF* collinear gene pairs, respectively. Each chromosome of the six Solanaceae species, except for the 11th chromosome of *C. annuum*, contains several genes that share collinear relationships with the *LbAP2/ERFs* in the *L. barbarum*, indicating that Solanaceae species exhibit a high degree of evolutionary conservation of putative *AP2/ERFs*.

Five gene duplication events were studied, revealing that among the 1,259 duplicated pairs in six Solanaceae species, DSD was the most prevalent mode, accounting for the maximum number of gene pairs, suggesting that the expansion of the *AP2/ERF* gene family was mainly associated with DSD. In contrast, only 25 PDs were identified in the *AP2/ERF* gene family (**Figure 2D**, **Supplementary Table S5**). For most *AP2/ERF* genes, the Ka/Ks was below 0.5, indicating strong purifying selection exerted on these genes (**Figure 2E**). In contrast, the gene pairs derived from PD and TD in *L. barbarum* had relatively higher Ka/Ks ratios compared with other types of duplicated gene pairs, suggesting that these specific gene pairs evolved at a higher rate than others.

### 3.3 Gene structure and conserved motif analysis of *LbAP2/ERFs* members

**Figure 3:**
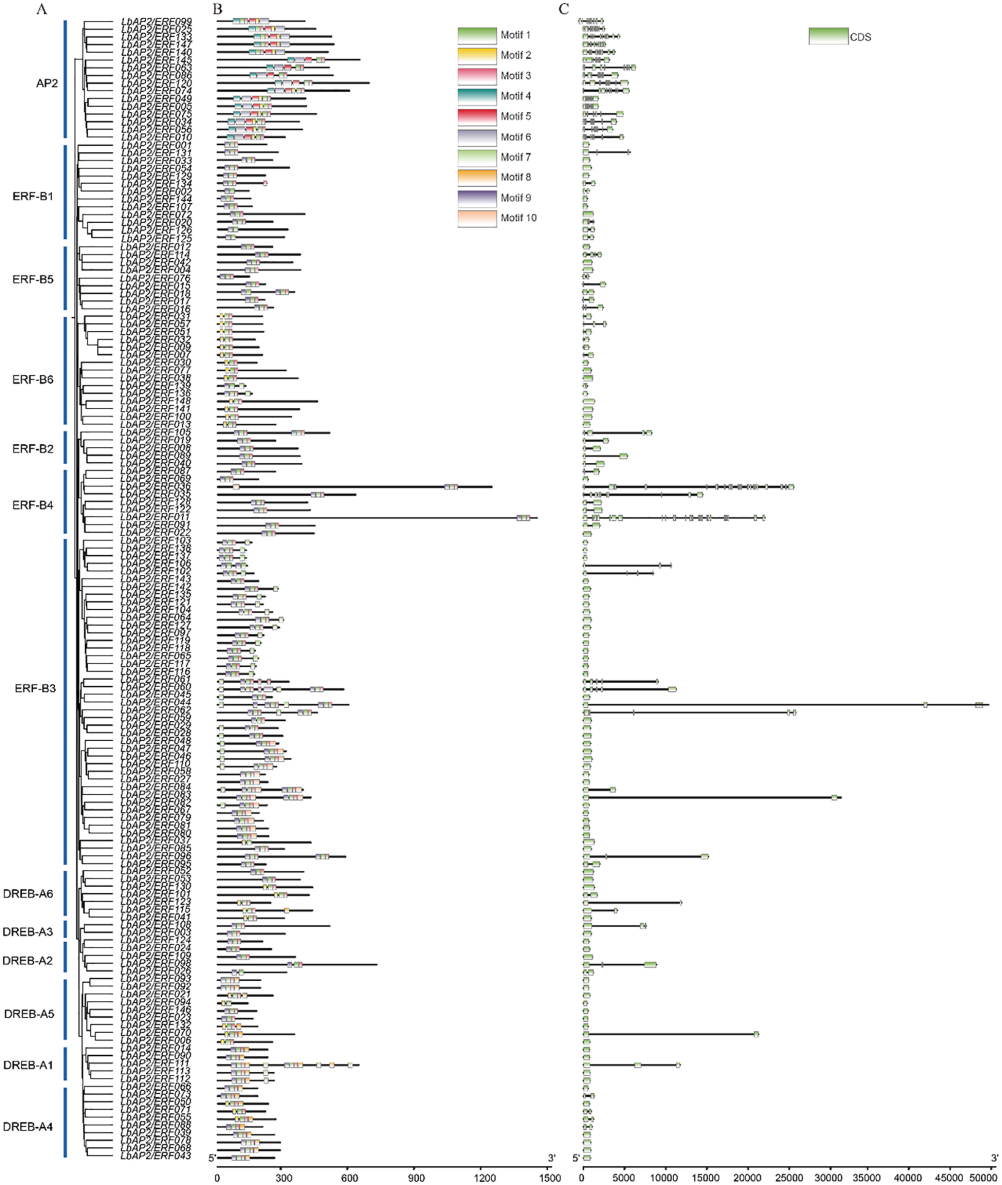
Phylogenetic relationship, conserved domains and gene structure of *LbAP2/ERFs* in *L. barbarum* (NQ). **(A)** Phylogenetic tree of LbAP2/ERF proteins. **(B)** Motif composition of LbAP2/ERF proteins, with different colors representing ten different motifs. **(C)** Exon-intron structures of *LbAP2/ERF* genes. Blue boxes indicate exon regions, and black lines indicate introns.

The conserved motifs of 148 LbAP2/ERF proteins were investigated using MEME software. Motif pattern analysis (**Figures 3A, B**) shows that each group features at least three distinct types of motifs. Notably, the AP2 subfamily, the DREB-A1 clade of the DREB subfamily, and the ERF-B3 clade of the ERF subfamily exhibit richly conserved motifs, exemplified by the intricate presence of five different types of motifs within *LbAP2/ERF049*. Additionally, a common trend of motif similarities can be observed across various groups. Gene structure analysis (**Figure 3C**) reveals that the *LbAP2/ERF* gene family consists of genes with varying numbers of exons, ranging from one to twenty-five. Subfamily AP2 displays diversity in exon composition, with genes in this subfamily have at least six exons. In contrast, DREB-A1 and DREB-A5 clades predominantly feature a streamlined exon structure, with most genes having a single exon, except for *LbAP2/ERF111* and *LbAP2/ERF070*, which have two exons. Notably, ERF-B4 shows significant variability in exon number, ranging from just one exon in *LbAP2/ERF069* to twenty-five exons in *LbAP2/ERF011*, making it the gene with the highest exon count in the entire gene pool. These results indicate that, with the exception of a few unique gene family members, genes within the same evolutionary branch exhibit similar conserved domains and intron distribution, suggesting a relative conservation of *AP2/ERFs* in *L. barbarum*.

### 3.4 Expression patterns analysis of *LbAP2/ERF* genes

**Figure 4:**
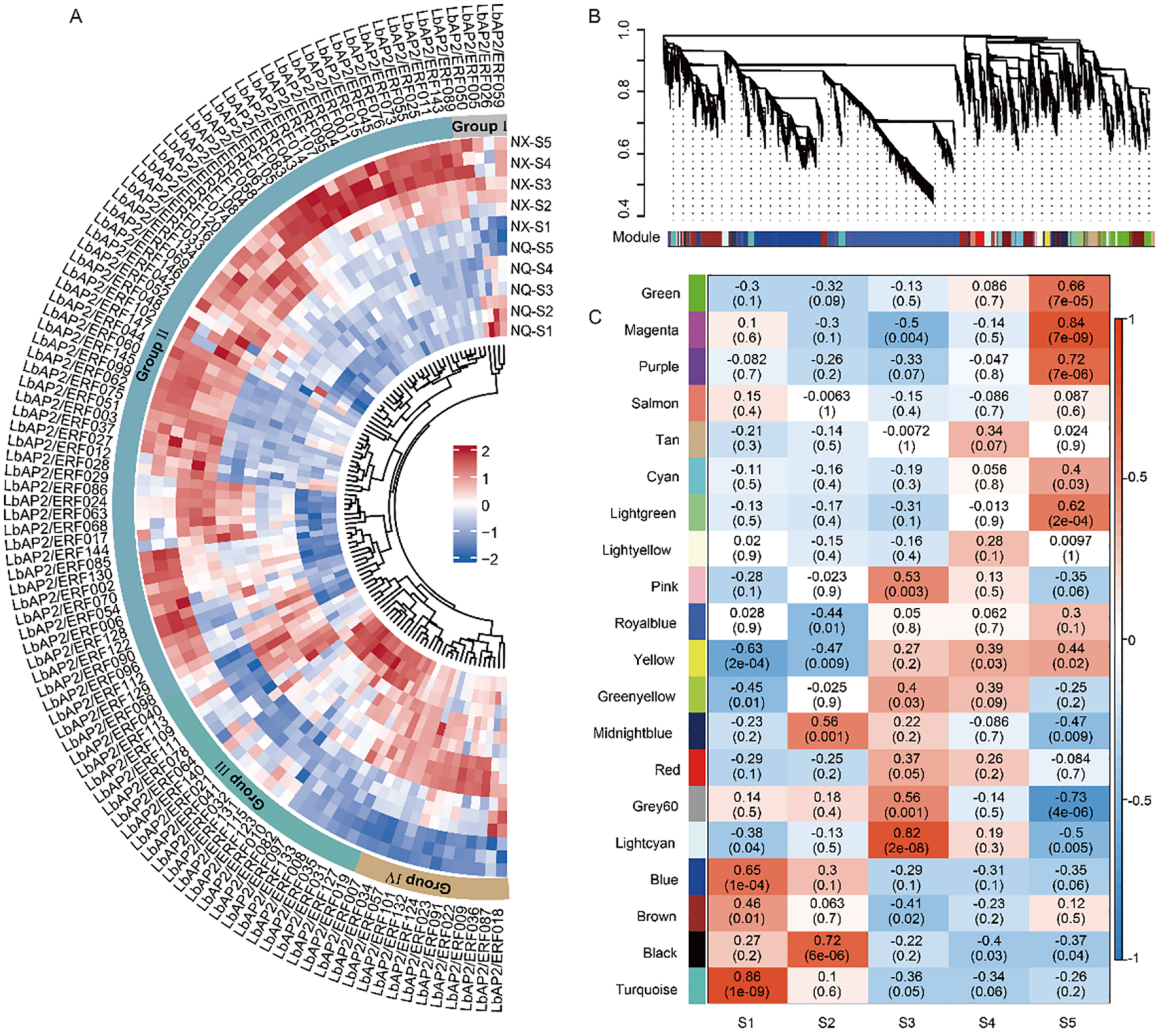
Expression analysis of *LbAP2/ERF* genes during wolfberry fruit development. **(A)** Heatmap of differentially expressed *LbAP2/ERF* genes in NQ and NX fruits at five developmental stages (S1-S5). Expression levels range from low (blue) to high (red). The central circular colors represent the clustering of *LbAP2/ERF* DEGs into Groups I (gray), II (light blue), III (light green), and I (light yellow). Scaled-TPM values were used to construct the heatmap based on hierarchical clustering analysis. **(B, C)** WGCNA of DEGs from NQ and NX fruits. **(B)** Hierarchical clustering tree showing 20 modules. Each leaf of the tree represents a DEG, and each major branch represents a module. **(C)** Correlation between modules and the fruit development process, with corresponding p-values in parentheses. The left color panel shows the modules, while the right color scale indicates the correlation between modules and the fruit development stage.

Transcriptomic profiles of two wolfberry varieties, NQ and NX, were analyzed across five fruit developmental stages (S1–S5). Using a cutoff of |log2 (fold change) | ≥ 1 and adjusted p-value < 0.05, we identified 23,348 differentially expressed genes (DEGs) between the two cultivars. Among these, 44 *LbAP2/ERF* genes showed low expression across all five stages in both varieties, suggesting that these genes might not be the key regulators of fruit development. The expression patterns of the differentially expressed *LbAP2/ERF* genes were clustered into four main groups, labeled I to IV (**Figure 4A**). Genes from different subfamilies of *LbAP2/ERF* were distributed across these groups. Most of the *LbAP2/ERF* genes exhibited similar expression patterns in both varieties, suggesting that these genes may play similar biological roles in fruit development across different cultivars. However, the *LbAP2/ERF* genes in Group II showed significantly higher expression levels in NX, suggesting that these genes may be involved in the formation of cultivar-specific substances. Group II and I genes were predominantly expressed in the later stages (S4 to S5), while Group I and III genes exhibited higher expression in the early stages (S1 to S2).

Weighted Gene Co-expression Network Analysis (WGCNA) based on 23,348 DEGs, which were assigned to 20 merged modules (**Figures 4B, C**; **Supplementary Table S6**). Eight modules, including green, magenta, purple, light green, light cyan, blue, black, and turquoise, showed significant correlations with fruit developmental stages (*p* < 0.001, correlation > 0.6). The blue, turquoise, black, and purple modules, with 37, 19, 3, and 1 *AP2/ERF* genes respectively, are rich in flavonoid-biosynthetic structural genes such as *PAL*, *C4H*, and *F3’H*. This suggests that these *AP2/ERF* genes in these modules may play a critical role in flavonoid synthesis during fruit development. In summary, *LbAP2/ERF* genes exhibit stage-specific expression patterns throughout fruit development and play crucial roles in regulating flavonoid synthesis and other aspects of fruit development.

### 3.5 Identification of *AP/ERF* genes involved in regulating flavonoid biosynthesis

Metabolomic analysis identified 180 differentially accumulated flavonoid metabolites (DAMs) in two wolfberry varieties, NQ and NX, at five developmental stages of fruit (fold change ≥ 2 or ≤ 0.5, VIP ≥ 1) (**Supplementary Table S7**). Within the WGCNA, a total of 34 *LbAP2/ERF* genes from the blue, black, purple, and turquoise modules exhibited significant correlations with various flavonoid metabolites. Notably, genes such as *LbAP2/ERF089*, *LbAP2/ERF011*, *LbAP2/ERF068*, and *LbAP2/ERF099* showed strong associations, suggesting that these genes may play a crucial role in flavonoid synthesis during wolfberry fruit development (**Supplementary Figure S3**).

### 3.6 Functional analysis of *LbAP2/ERF089* in flavonoid biosynthesis

**Figure 5:**
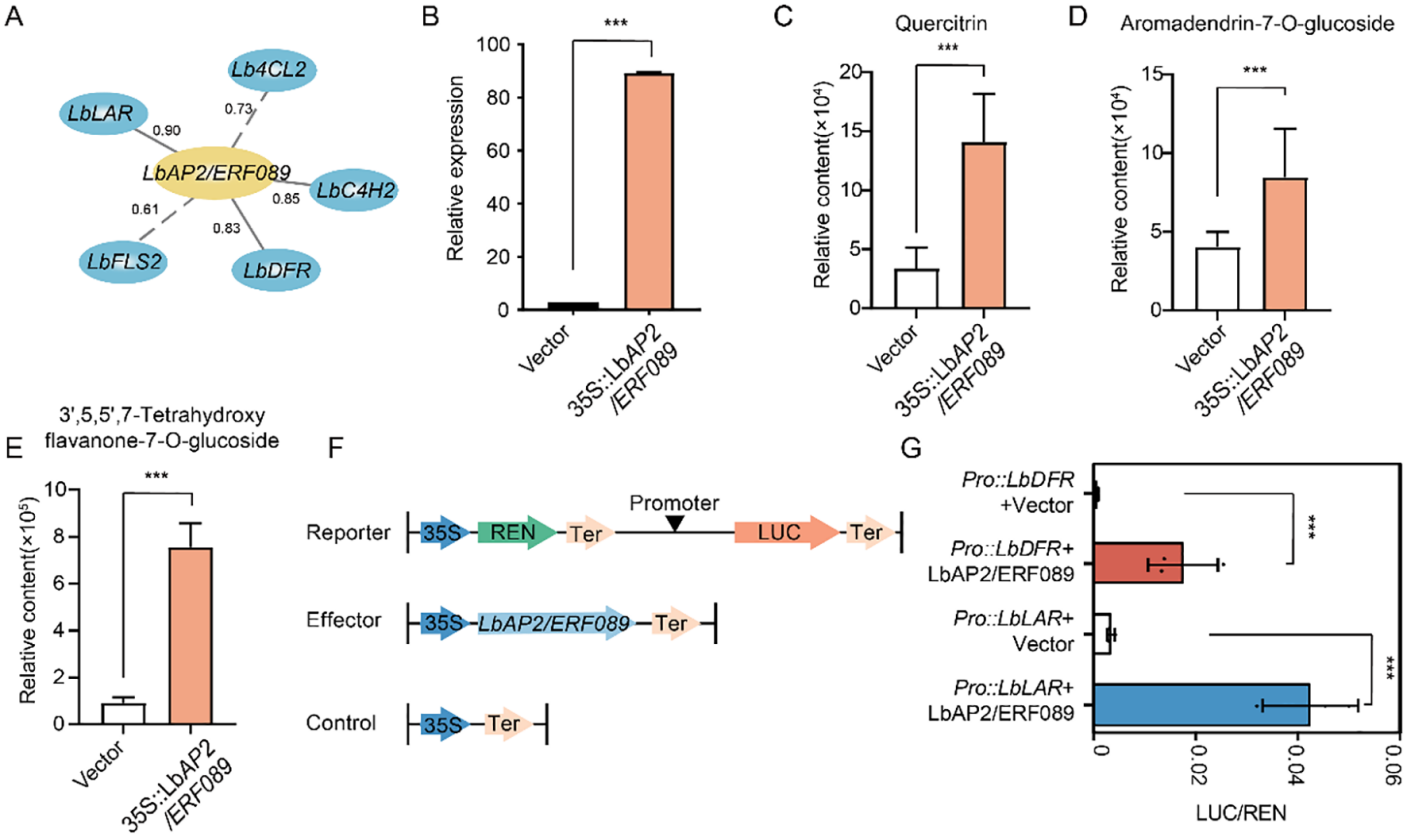
Functional analysis of *LbAP2/ERF089* in flavonoid synthesis. **(A)** Correlation between *LbAP2/ERF089* and structural genes involved in flavonoid biosynthesis in NQ and NX fruits. The gray solid line indicates correlations above 0.8, while dashes represent correlations below 0.8. **(B)** Transcript levels of *LbAP2/ERF089* in NQ leaves with transient overexpression. The relative expression levels of *LbAP2/ERF089* were quantified by qRT-PCR. **(C–E)** Flavonoid accumulation profiles in NQ leaves after *LbAP2/ERF089* transient expression. **(C)** Quercitrin, **(D)** Aromadendrin-7-O-glucoside, and **(E)** 3’,5,5’,7-Tetrahydroxyflavone content in the leaves of *L. barbarum* with transient expression of *LbAP2/ERF089*. **(F)** Schematic diagram of the effector and reporter plasmids used in the transient assay in leaf epidermal cells of *N. benthamiana*. REN, Renilla luciferase; LUC, firefly luciferase. **(G)** *LbAP2/ERF089* activates the transcription of *LbLAR* and *LbDFR* (cloned from NQ). *N. benthamiana* leaves were infiltrated with different combinations of effectors and reporters. The LUC activity was normalized to the REN activity as an internal control. Student’s t-test was used for comparisons (***, p < 0.001).

Among the 34 *AP2/ERF* genes, we selected *LbAP2/ERF089*, which showed significant associations with multiple flavonoid synthesis genes and metabolites, to investigate its potential role in flavonoid biosynthesis (**Figure 5A**, **Supplementary Table S6**). An overexpression vector of *LbAP2/ERF089* was constructed and transiently expressed in the leaves of *L. barbarum*. The accumulation of flavonoid metabolites, including quercitrin, aromadendrin-7-O-glucoside, 3’,5,5’,7-tetrahydroxyflavone, and twenty other flavonoid metabolites, was significantly higher in the plants overexpressing *LbAP2/ERF089* compared to those with the empty vector (**Figures 5B–E**, **Supplementary Table S8**), indicating that overexpression of *LbAP2/ERF089* promotes flavonoid accumulation.

To further confirm the results, we cotransfected the effector *LbAP2/ERF089* (35S::*LbAP2/ERF089*) with the reporter containing the *LbLAR*, or *LbDFR* promoter driving luciferase (*ProLbLAR::LUC*, or *ProLbDFR::LUC*) in leaf epidermal cells of *N. benthamiana*, and the relative luciferase (LUC) activity was measured (**Figure 5F**). The results showed that co-expression with *LbAP2/ERF089* (35S::*LbAP2/ERF089*) significantly increased the luciferase activity of *ProLbLAR::LUC* and *ProLbDFR::LUC* (**Figure 5G**), indicating that *LbAP2/ERF089* functions as a transcriptional activator of *LbLAR* and *LbDFR* expression, thereby enhancing flavonoid and anthocyanin synthesis.

## 4 Discussion

The AP2/ERF genes comprise a large family of TFs that have been identified in various plants such as *Zingiber officinale* (Xing et al., 2021), *Rosa chinensis* (Li et al., 2020a), and *Fagopyrum tataricum* (Liu et al., 2019b). This study identified 148 potential *AP2/ERF* genes in *L. barbarum* based on published genome data. The number of AP2/ERF transcription factors and their subfamily members varies among plant species, with ERF and DREB classes representing more than 50% of the AP2/ERF transcription factors (Wu et al., 2015; Liu et al., 2023). In contrast, the number of Soloist-like AP2/ERF transcription factors is minimal, with most plant species possessing only one or none, as observed in Arabidopsis (Sakuma et al., 2002; Nakano et al., 2006). Similarly, the Soloist subfamily is absent in the AP2/ERF family of *L. barbarum*, and it is noteworthy that the RAV subfamily is also missing, indicating that this group has evolved or been lost in *L. barbarum* during evolution.

Gene duplication is a major source of new genes in evolution (Rezaee et al., 2020; Faraji et al., 2021). In the genome of *L. barbarum*, WGD events are the primary cause of gene family expansion (Cao et al., 2021). In most plants, WGD or SD plays a dominant role in the expansion of the *AP2/ERF* gene family, as evidenced by studies in pear (Li et al., 2018) and pumpkin (Li et al., 2022). We found that DSD may be the primary driver of *LbAP2/ERF* gene family’s amplification. This indicates that the *AP2/ERF* gene family in *L. barbarum* may exhibit a distinct evolutionary pattern.

The *AP2/ERF* family is a large class of transcription factors involved in various plant life processes (Ohme-Takagi and Shinshi, 1995), participating in both primary and secondary metabolism, such as artemisinin, flavonoid, and carotene metabolism (Zhao et al., 2019; Han et al., 2022; Zhang et al., 2023). The tissue-specific expression of *AP2/ERF* genes may influence the synthesis of secondary metabolites by regulating transcription processes (Shoji and Yuan, 2021). Consistent with previous studies, there are significant differences in the types and levels of flavonoid metabolites between NX and NQ (Du et al., 2024). We found that the expression of *AP2/ERF* genes belonging to Groups II reflects the specificity of varieties, indicating that these genes may be involved in regulating the synthesis of flavonoids in different varieties. Through integrated transcriptomic and metabolomic analyses, we identified 34 *LbAP2/ERF* genes associated with flavonoid metabolism. However, most of these genes are expressed at low levels during fruit development. Previous research has been confirmed that *AP2/ERF* genes are often rapidly and continuously induced under stress conditions, while their responses to other processes are relatively slower (Zhao et al., 2022). In addition, *AP2/ERF* genes play important roles in regulating the synthesis of flavonoid compounds (An et al., 2018; Yao et al., 2017; Ni et al., 2019, 2021; Mo et al., 2022). In this study, *LbAP2/ERF089* was found to regulate flavonoid metabolism in wolfberries by interacting with *LbLAR* and *LbDFR*, consistent with previous research linking *DFR* and *LAR* to anthocyanin synthesis (Shan et al., 2020). Notably, overexpression of *LbAP2/ERF089* also promoted accumulation of phenols and amino acids, with regulatory mechanisms needing further exploration.

In summary, we identified a total of 148 high-confidence AP2/ERF transcription factors in *L. barbarum*, including several key candidates implicated in flavonoid biosynthesis, such as *LbAP2/ERF089*, *LbAP2/ERF068*, and *LbAP2/ERF099*. Notably, *LbAP2/ERF089* was functionally validated as a positive regulator of flavonoid accumulation in wolfberry. These findings establish a putative AP2/ERF-mediated regulatory network governing flavonoid metabolism in *L. barbarum*, providing a valuable genetic resource for future molecular breeding efforts aimed at enhancing flavonoid content.

## 5 Conclusion

In this study, we identified 148 *LbAP2/ERF* genes, with dispersed duplication serves as the primary driver of their amplification. Expression profiling of these genes during fruit development revealed distinct temporal expression patterns, indicating their diverse roles in wolfberry fruit biology. Additionally, several *LbAP2/ERF* genes involved in flavonoid biosynthesis were identified. Further research showed that *LbAP2/ERF089* functions as a positive regulator of flavonoid biosynthesis. Taken together, our findings identify AP2/ERF transcription factors involved in regulating wolfberry flavonoid and anthocyanin biosynthesis and provide new insights into their roles in transcriptional regulatory mechanisms.

## Data Availability

The datasets presented in this study can be found in online repositories. The names of the repository/repositories and accession number(s) can be found in the article/**Supplementary Material**.

## References

[B1] Amagase H. Farnsworth N. R. (2011). A review of botanical characteristics, phytochemistry, clinical relevance in efficacy and safety of *Lycium barbarum* fruit (Goji). Food Res. Int. 44, 1702–1717. doi: 10.1016/j.foodres.2011.03.027

[B2] Amagase H. Sun B. X. Borek C. (2009a). *Lycium barbarum* (goji) juice improves *in vivo* antioxidant biomarkers in serum of healthy adults. Nutr. Res. 29, 19–25. doi: 10.1016/j.nutres.2008.11.005, PMID: 19185773

[B3] Amagase H. Sun B. X. Nance D. M. (2009b). Immunomodulatory effects of a standardized *Lycium barbarum* fruit juice in Chinese older healthy human subjects. J. Medicinal Food 12, 1159–1165. doi: 10.1089/jmf.2008.0300, PMID: 19857084

[B4] Amato A. Cavallini E. Zenoni S. Finezzo L. Begheldo M. Ruperti B.. (2017). A grapevine TTG2-Like WRKY transcription factor is involved in regulating vacuolar transport and flavonoid biosynthesis. Front. Plant Sci. 7. doi: 10.3389/fpls.2016.01979, PMID: 28105033 PMC5214514

[B5] An J. P. Wang X. F. Li Y. Y. Song L. Q. Zhao L. L. You C. X.. (2018). EIN3-LIKE1, MYB1, and ETHYLENE RESPONSE FACTOR3 act in a regulatory loop that synergistically modulates ethylene biosynthesis and anthocyanin accumulation. Plant Physiol. 178, 808–823. doi: 10.1104/pp.18.00068, PMID: 29925585 PMC6181056

[B6] Bondia-Pons I. Savolainen O. Törrönen R. Martinez J. A. Poutanen K. Hanhineva K. (2014). Metabolic profiling of Goji berry extracts for discrimination of geographical origin by non-targeted liquid chromatography coupled to quadrupole time-of-flight mass spectrometry. Food Res. Int. 63, 132–138. doi: 10.1016/j.foodres.2014.01.067

[B7] Cao Y. L. Li Y. L. Fan Y. F. Li Z. Yoshida K. Wang J. Y.. (2021). Wolfberry genomes and the evolution of *Lycium* (Solanaceae). Commun. Biol. 4, 671. doi: 10.1038/s42003-021-02152-8, PMID: 34083720 PMC8175696

[B8] Chen C. J. Chen H. Zhang Y. Thomas H. R. Frank M. H. He Y. H.. (2020). TBtools: an integrative toolkit developed for interactive analyses of big biological data. Mol. Plant 13, 1194–1202. doi: 10.1016/j.molp.2020.06.009, PMID: 32585190

[B9] Chen S. F. Zhou Y. Q. Chen Y. R. Gu J. (2018). Fastp: an ultra-fast all-in-one FASTQ preprocessor. Bioinformatics 34, 884–890. doi: 10.1093/bioinformatics/bty560, PMID: 30423086 PMC6129281

[B10] Du Y. W. Ma H. Y. Liu Y. Y. Gong R. Lan Y. Zhao J. H.. (2024). Major quality regulation network of flavonoid synthesis governing the bioactivity of black wolfberry. New Phytol. 242, 558–575. doi: 10.1111/nph.19602, PMID: 38396374

[B11] Emms D. M. Kelly S. (2019). OrthoFinder: phylogenetic orthology inference for comparative genomics. Genome Biol. 20, 238. doi: 10.1186/s13059-019-1832-y, PMID: 31727128 PMC6857279

[B12] Faraji S. Heidari P. Amouei H. Filiz E. Poczai P. (2021). Investigation and computational analysis of the Sulfotransferase (SOT) gene family in Potato (*Solanum tuberosum*): insights into sulfur adjustment for proper development and stimuli responses. Plants-Basel 10 (12), 2597. doi: 10.3390/plants10122597, PMID: 34961068 PMC8707064

[B13] Gao Q. Q. Song W. L. Li X. Xiang C. F. Chen G. Xiang G. S.. (2022). Genome-wide identification of bHLH transcription factors: discovery of a candidate regulator related to flavonoid biosynthesis in *Erigeron breviscapus* . Front. Plant Sci. 13. doi: 10.3389/fpls.2022.977649, PMID: 36186051 PMC9515989

[B14] Gao J. Zhang Y. X. Li Z. G. Liu M. C. (2020). Role of ethylene response factors (ERFs) in fruit ripening. Food Qual. Saf. 4, 15–19. doi: 10.1093/fqsafe/fyz042

[B15] Han D. Huang B. C. Li Y. C. Dang Q. Y. Fan L. M. Nie J. Y.. (2022). The MdAP2–34 modulates flavonoid accumulation in apple (*Malus domestica* Borkh.) by regulating *MdF3’H* . Postharvest Biol. Technol. 192, 111994. doi: 10.1016/j.postharvbio.2022.111994

[B16] Ito K. Murphy D. (2013). Application of *ggplot2* to pharmacometric graphics. CPT Pharmacometrics Syst. Pharmacol. 2, e79. doi: 10.1038/psp.2013.56, PMID: 24132163 PMC3817376

[B17] Jiang L. F. (2014). Preparation and antioxidant activity of *Lycium barbarum* oligosaccharides. Carbohydr. Polymers 99, 646–648. doi: 10.1016/j.carbpol.2013.08.084, PMID: 24274555

[B18] Jin M. L. Huang Q. S. Zhao K. Shang P. (2013). Biological activities and potential health benefit effects of polysaccharides isolated from *Lycium barbarum* L. Int. J. Biol. Macromolecules 54, 16–23. doi: 10.1016/j.ijbiomac.2012.11.023, PMID: 23200976

[B19] Kim D. Landmead B. Salzberg S. L. (2015). HISAT: a fast spliced aligner with low memory requirements. Nat. Methods 12, 357–U121. doi: 10.1038/Nmeth.3317, PMID: 25751142 PMC4655817

[B20] Kim S.-H. Lee J.-R. Hong S.-T. Yoo Y.-K. An G. Kim S.-R. (2003). Molecular cloning and analysis of anthocyanin biosynthesis genes preferentially expressed in apple skin. Plant Sci. 165, 403–413. doi: 10.1016/s0168-9452(03)00201-2

[B21] Kumar S. Stecher G. Li M. Knyaz C. Tamura K. (2018). MEGA X: molecular evolutionary genetics analysis across computing platforms. Mol. Biol. Evol. 35, 1547–1549. doi: 10.1093/molbev/msy096, PMID: 29722887 PMC5967553

[B22] Langfelder P. Horvath S. (2008). WGCNA: an R package for weighted correlation network analysis. BMC Bioinf. 9, 559. doi: 10.1186/1471-2105-9-559, PMID: 19114008 PMC2631488

[B23] Li Y. Chen Y. Zhou L. You S. J. Deng H. Chen Y.. (2020b). MicroTom metabolic network: rewiring tomato metabolic regulatory network throughout the growth cycle. Mol. Plant 13, 1203–1218. doi: 10.1016/j.molp.2020.06.005, PMID: 32561360

[B24] Li D. Liu X. Shu L. Zhang H. Zhang S. Song Y.. (2020a). Global analysis of the AP2/ERF gene family in rose (*Rosa chinensis*) genome unveils the role of *RcERF099* in Botrytis resistance. BMC Plant Biol. 20, 533. doi: 10.1186/s12870-020-02740-6, PMID: 33228522 PMC7684944

[B25] Li X. L. Tao S. Wei S. W. Ming M. L. Huang X. S. Zhang S. L.. (2018). The mining and evolutionary investigation of *AP2/ERF* genes in pear (*Pyrus*). BMC Plant Biol. 18, 46. doi: 10.1186/s12870-018-1265-x, PMID: 29558898 PMC5859658

[B26] Li Q. F. Zhang L. Chen P. W. Wu C. H. Zhang H. X. Yuan J. P.. (2022). Genome-wide identification of *APETALA2/ETHYLENE RESPONSIVE FACTOR* transcription factors in *Cucurbita moschata* and their involvement in ethylene response. Front. Plant Sci. 13. doi: 10.3389/fpls.2022.847754, PMID: 35371131 PMC8965380

[B27] Liao Y. Smyth G. K. Shi W. (2014). FeatureCounts: an efficient general purpose program for assigning sequence reads to genomic features. Bioinformatics 30, 923–930. doi: 10.1093/bioinformatics/btt656, PMID: 24227677

[B28] Liu H. L. Su B. B. Zhang H. Gong J. X. Zhang B. X. Liu Y. L.. (2019a). Identification and functional analysis of a flavonol synthase gene from grape hyacinth. Molecules 24 (8), 1579. doi: 10.3390/molecules24081579, PMID: 31013599 PMC6514955

[B29] Liu M. Y. Sun W. J. Ma Z. T. Zheng T. R. Huang L. Wu Q.. (2019b). Genome-wide investigation of the *AP2/ERF* gene family in tartary buckwheat (*Fagopyrum Tataricum*). BMC Plant Biol. 19, 84. doi: 10.1186/s12870-019-1681-6, PMID: 31185913 PMC6558689

[B30] Liu X. J. Zhou G. Y. Chen S. S. Jia Z. Z. Zhang S. Q. Ren M. J.. (2023). Genome-wide analysis of the *AP2/ERF* gene family in *Tritipyrum* and the response of *TtERF_B2–50* in salt-tolerance. BMC Genomics 24 (1), 541. doi: 10.1186/s12864-023-09585-x, PMID: 37704958 PMC10498623

[B31] Livak K. J. Schmittgen T. D. (2001). Analysis of relative gene expression data using real-time quantitative PCR and the 2(-delta delta C(T)) method. Methods 25, 402–408. doi: 10.1006/meth.2001.1262, PMID: 11846609

[B32] Miean K. H. Mohamed S. (2001). Flavonoid (myricetin, quercetin, kaempferol, luteolin, and apigenin) content of edible tropical plants. J. Agric. Food Chem. 49, 3106–3112. doi: 10.1021/jf000892m, PMID: 11410016

[B33] Minh B. Q. Schmidt H. A. Chernomor O. Schrempf D. Woodhams M. D. von Haeseler A.. (2020). IQ-TREE 2: new models and efficient methods for phylogenetic inference in the genomic era. Mol. Biol. Evol. 37, 2461–2461. doi: 10.1093/molbev/msaa131, PMID: 32556291 PMC7403609

[B34] Mizoi J. Shinozaki K. Yamaguchi-Shinozaki K. (2012). AP2/ERF family transcription factors in plant abiotic stress responses. Biochim. Et Biophys. Acta-Gene Regul. Mech. 1819, 86–96. doi: 10.1016/j.bbagrm.2011.08.004, PMID: 21867785

[B35] Mo R. Han G. Zhu Z. Essemine J. Dong Z. Li Y.. (2022). The ethylene response factor ERF5 regulates anthocyanin biosynthesis in ‘Zijin’ mulberry fruits by interacting with *MYBA* and *F3H* genes. Int. J. Mol. Sci. 23, 7615. doi: 10.3390/ijms23147615, PMID: 35886963 PMC9318412

[B36] Nakano T. Suzuki K. Fujimura T. Shinshi H. (2006). Genome-wide analysis of the *ERF* gene family in *Arabidopsis* and rice^[W]^ . Plant Physiol. 140, 411–432. doi: 10.1104/pp.105.073783, PMID: 16407444 PMC1361313

[B37] Ni J. Bai S. Zhao Y. Qian M. Tao R. Yin L.. (2019). Ethylene response factors Pp4ERF24 and Pp12ERF96 regulate blue light-induced anthocyanin biosynthesis in ‘Red Zaosu’ pear fruits by interacting with MYB114. Plant Mol. Biol. 99, 67–78. doi: 10.1007/s11103-018-0802-1, PMID: 30539403

[B38] Ni J. Premathilake A. T. Gao Y. Yu W. Tao R. Teng Y.. (2021). Ethylene-activated PpERF105 induces the expression of the repressor-type R2R3-MYB gene *PpMYB140* to inhibit anthocyanin biosynthesis in red pear fruit. he Plant J. 105, 167–181. doi: 10.1111/tpj.15049, PMID: 33111423

[B39] Ohme-Takagi M. Shinshi H. (1995). Ethylene-inducible DNA binding proteins that interact with an ethylene-responsive element. Plant Cell 7, 173–182. doi: 10.1105/tpc.7.2.173, PMID: 7756828 PMC160773

[B40] Qiao X. Li Q. Yin H. Qi K. Li L. Wang R.. (2019). Gene duplication and evolution in recurring polyploidization-diploidization cycles in plants. Genome Biol. 20, 38. doi: 10.1186/s13059-019-1650-2, PMID: 30791939 PMC6383267

[B41] Rezaee S. Ahmadizadeh M. Heidari P. (2020). Genome-wide characterization, expression profiling, and post-transcriptional study of *GASA* gene family. Gene Rep. 20, 100795. doi: 10.1016/i.genrep.2020.100795

[B42] Riechmann J. L. Meyerowitz E. M. (1998). The *AP2/EREBP* family of plant transcription factors. Biol. Chem. 379, 633–646. doi: 10.1515/bchm.1998.379.6.633, PMID: 9687012

[B43] Sainz M. B. Grotewold E. Chandler V. L. (1997). Evidence for direct activation of an anthocyanin promoter by the maize C1 protein and comparison of DNA binding by related Myb domain proteins. Plant Cell 9, 611–625. doi: 10.1105/tpc.9.4.611, PMID: 9144964 PMC156943

[B44] Sakuma Y. Liu Q. Dubouzet J. G. Abe H. Shinozaki K. Yamaguchi-Shinozaki K. (2002). DNA-binding specificity of the ERF/AP2 domain of Arabidopsis DREBs, transcription factors involved in dehydration-and cold-inducible gene expression. Biochem. Biophys. Res. Commun. 290, 998–1009. doi: 10.1006/bbrc.2001.6299, PMID: 11798174

[B45] Shan X. Li Y. Yang S. Yang Z. Qiu M. Gao R.. (2020). The spatio-temporal biosynthesis of floral flavonols is controlled by differential phylogenetic MYB regulators in *Freesia hybrida* . New Phytol. 228, 1864–1879. doi: 10.1111/nph.16818, PMID: 32696979

[B46] Shannon P. Markiel A. Ozier O. Baliga N. S. Wang J. T. Ramage D.. (2003). Cytoscape: a software environment for integrated models of biomolecular interaction networks. Genome Res. 13, 2498–2504. doi: 10.1101/gr.1239303, PMID: 14597658 PMC403769

[B47] Shoji T. Yuan L. (2021). ERF gene clusters: working together to regulate metabolism. Trends Plant Sci. 26, 23–32. doi: 10.1016/j.tplants.2020.07.015, PMID: 32883605

[B48] Sparvoli F. Martin C. Scienza A. Gavazzi G. Tonelli C. (1994). Cloning and molecular analysis of structural genes involved in flavonoid and stilbene biosynthesis in grape (*Vitis vinifera* L.). Plant Mol. Biol. 24, 743–755. doi: 10.1007/bf00029856, PMID: 8193299

[B49] Stracke R. De Vos R. C. Bartelniewoehner L. Ishihara H. Sagasser M. Martens S.. (2009). Metabolomic and genetic analyses of flavonol synthesis in *Arabidopsis thaliana* support the *in vivo* involvement of leucoanthocyanidin dioxygenase. Planta 229, 427–445. doi: 10.1007/s00425-008-0841-y, PMID: 18998159

[B50] Stracke R. Ishihara H. Huep G. Barsch A. Mehrtens F. Niehaus K.. (2007). Differential regulation of closely related R2R3-MYB transcription factors controls flavonol accumulation in different parts of the *Arabidopsis thaliana* seedling. Plant J. 50, 660–677. doi: 10.1111/j.1365-313X.2007.03078.X, PMID: 17419845 PMC1976380

[B51] Tsai C. J. Harding S. A. Tschaplinski T. J. Lindroth R. L. Yuan Y. (2006). Genome-wide analysis of the structural genes regulating defense phenylpropanoid metabolism in *Populus* . New Phytol. 172, 47–62. doi: 10.1111/j.1469-8137.2006.01798.x, PMID: 16945088

[B52] Untergasser A. Cutcutache I. Koressaar T. Ye J. Faircloth B. C. Remm M.. (2012). Primer3–new capabilities and interfaces. Nucleic Acids Res. 40, e115. doi: 10.1093/nar/gks596, PMID: 22730293 PMC3424584

[B53] Wang C. Dong Y. Zhu L. Wang L. Yan L. Wang M.. (2020). Comparative transcriptome analysis of two contrasting wolfberry genotypes during fruit development and ripening and characterization of the *LrMYB1* transcription factor that regulates flavonoid biosynthesis. BMC Genomics 21, 295. doi: 10.1186/s12864-020-6663-4, PMID: 32272876 PMC7147035

[B54] Wang L. Guo D. Zhao G. Wang J. Zhang S. Wang C.. (2022). Group IIc WRKY transcription factors regulate cotton resistance to *Fusarium oxysporum* by promoting GhMKK2-mediated flavonoid biosynthesis. New Phytol. 236, 249–265. doi: 10.1111/nph.18329, PMID: 35727190

[B55] Wang Z. Song G. Zhang F. Shu X. Wang N. (2023). Functional characterization of AP2/ERF transcription factors during flower development and anthocyanin biosynthesis related candidate genes in *Lycoris* . Int. J. Mol. Sci. 24, 14464. doi: 10.3390/ijms241914464, PMID: 37833913 PMC10572147

[B56] Wang Y. Tang H. Debarry J. D. Tan X. Li J. Wang X.. (2012). *MCScanX*: a toolkit for detection and evolutionary analysis of gene synteny and collinearity. Nucleic Acids Res. 40, e49. doi: 10.1093/nar/gkr1293, PMID: 22217600 PMC3326336

[B57] Wang L. M. Zhang J. Dong X. Y. Fu Z. Z. Jiang H. Zhang H. C. (2018). Identification and functional analysis of anthocyanin biosynthesis genes in *Phalaenopsis* hybrids. Biol. Plantarum 62, 45–54. doi: 10.1007/s10535-017-0763-2

[B58] Wang D. Zhang Y. Zhang Z. Zhu J. Yu J. (2010). KaKs_Calculator 2.0: a toolkit incorporating gamma-series methods and sliding window strategies. Genomics Proteomics Bioinf. 8, 77–80. doi: 10.1016/s1672-0229(10)60008-3, PMID: 20451164 PMC5054116

[B59] Wheeler T. J. Eddy S. R. (2013). Nhmmer: DNA homology search with profile HMMs. Bioinformatics 29, 2487–2489. doi: 10.1093/bioinformatics/btt403, PMID: 23842809 PMC3777106

[B60] Winkel-Shirley B. (2001). Flavonoid biosynthesis. A colorful model for genetics, biochemistry, cell biology, and biotechnology. Plant Physiol. 126, 485–493. doi: 10.1104/pp.126.2.485, PMID: 11402179 PMC1540115

[B61] Wu H. Lv H. Li L. Liu J. Mu S. Li X.. (2015). Genome-wide analysis of the AP2/ERF transcription factors family and the expression patterns of *DREB* genes in moso bamboo (*Phyllostachys edulis*). PloS One 10, e0126657. doi: 10.1371/journal.pone.0126657, PMID: 25985202 PMC4436012

[B62] Xing H. Jiang Y. Zou Y. Long X. Wu X. Ren Y.. (2021). Genome-wide investigation of the AP2/ERF gene family in ginger: evolution and expression profiling during development and abiotic stresses. BMC Plant Biol. 21, 561. doi: 10.1186/s12870-021-03329-3, PMID: 34823471 PMC8620233

[B63] Yadav M. L. Roychoudhury B. (2018). Handling missing values:a study of popular imputation packages in R. Knowledge-Based Syst. 160, 104–118. doi: 10.1016/j.knosys.2018.06.012

[B64] Yao G. Ming M. Allan A. C. Gu C. Li L. Wu X.. (2017). Map-based cloning of the pear gene *MYB114* identifies an interaction with other transcription factors to coordinately regulate fruit anthocyanin biosynthesis. Plant J. 92, 437–451. doi: 10.1111/tpj.13666, PMID: 28845529

[B65] Ye G. Zheng Z. Zhou Y. Pu X. Su W. Guo H.. (2021). The MYB transcription factor *LrAN2*, from *Lycium ruthenicum*, led to enhanced accumulation of anthocyanins and modified profile of the total glycoalkaloids in potato. Plant Cell Tissue Organ Culture (PCTOC) 147, 519–528. doi: 10.1007/s11240-021-02144-w

[B66] Zeng S. Wu M. Zou C. Liu X. Shen X. Hayward A.. (2014). Comparative analysis of anthocyanin biosynthesis during fruit development in two *Lycium* species. Physiologia Plantarum 150, 505–516. doi: 10.1111/ppl.12131, PMID: 24661321

[B67] Zhang J. Zhao H. Chen L. Lin J. Wang Z. Pan J.. (2023). Multifaceted roles of WRKY transcription factors in abiotic stress and flavonoid biosynthesis. Front. Plant Sci. 14. doi: 10.3389/fpls.2023.1303667, PMID: 38169626 PMC10758500

[B68] Zhao M. Haxim Y. Liang Y. Qiao S. Gao B. Zhang D.. (2022). Genome-wide investigation of *AP2/ERF* gene family in the desert legume *Eremosparton songoricum*: identification, classification, evolution, and expression profiling under drought stress. Front. Plant Sci. 13. doi: 10.3389/fpls.2022.885694, PMID: 36035670 PMC9413063

[B69] Zhao M. Li J. Zhu L. Chang P. Li L. Zhang L. (2019). Identification and characterization of MYB-bHLH-WD40 regulatory complex members controlling anthocyanidin biosynthesis in blueberry fruits development. Genes (Basel) 10 (7), 496. doi: 10.3390/genes10070496, PMID: 31261791 PMC6678982

[B70] Zhao C. Liu X. Gong Q. Cao J. Shen W. Yin X.. (2021a). Three AP2/ERF family members modulate flavonoid synthesis by regulating type IV chalcone isomerase in citrus. Plant Biotechnol. J. 19, 671–688. doi: 10.1111/pbi.13494, PMID: 33089636 PMC8051604

[B71] Zhao J. Sun C. Shi F. Ma S. Zheng J. Du X.. (2021b). Comparative transcriptome analysis reveals sesquiterpenoid biosynthesis among 1-, 2- and 3-year old *Atractylodes chinensis* . BMC Plant Biol. 21, 354. doi: 10.1186/s12870-021-03131-1, PMID: 34315414 PMC8314494

[B72] Zhao J. Xu Y. Li H. An W. Yin Y. Wang B.. (2024). Metabolite-based genome-wide association studies enable the dissection of the genetic bases of flavonoids, betaine and spermidine in wolfberry (*Lycium*). Plant Biotechnol. J. 22, 1435–1452. doi: 10.1111/pbi.14278, PMID: 38194521 PMC11123438

[B73] Zhao J. Xu Y. Li H. Zhu X. Yin Y. Zhang X.. (2023). *ERF5.1* modulates carotenoid accumulation by interacting with *CCD4.1* in *Lycium* . Horticulture Res. 10, uhad230. doi: 10.1093/hr/uhad230, PMID: 38143484 PMC10745278

[B74] Zhong C. Tang Y. Pang B. Li X. Yang Y. Deng J.. (2020). The R2R3-MYB transcription factor GhMYB1a regulates flavonol and anthocyanin accumulation in *Gerbera hybrida* . Horticulture Res. 7, 78. doi: 10.1038/s41438-020-0296-2, PMID: 32435501 PMC7237480

