# Supplementary material for: Functional analysis of AP2/ERF family members in flavonoid biosynthesis of wolfberry
Source: Front Plant Sci. 2025 Sep 2;16:1632482. doi: 10.3389/fpls.2025.1632482 (PMC12436402; doi:10.3389/fpls.2025.1632482)
Supplement: Supplementary file 1 [file DataSheet1.docx]

**Supplementary information**


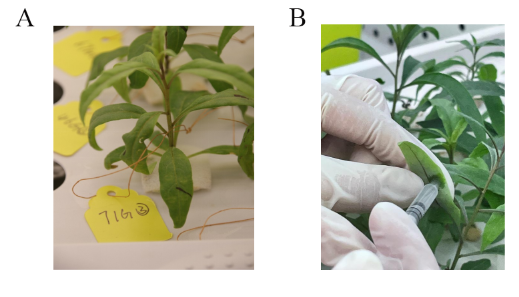


**Supplementary Figure 1.** Hydroponic NQ seedlings and agroinfiltration. (A) One-month-old NQ hydroponic seedlings cultured in MS solution. (B) Needleless syringe injecting Agrobacterium suspension into the abaxial side of NQ seedling leaves.


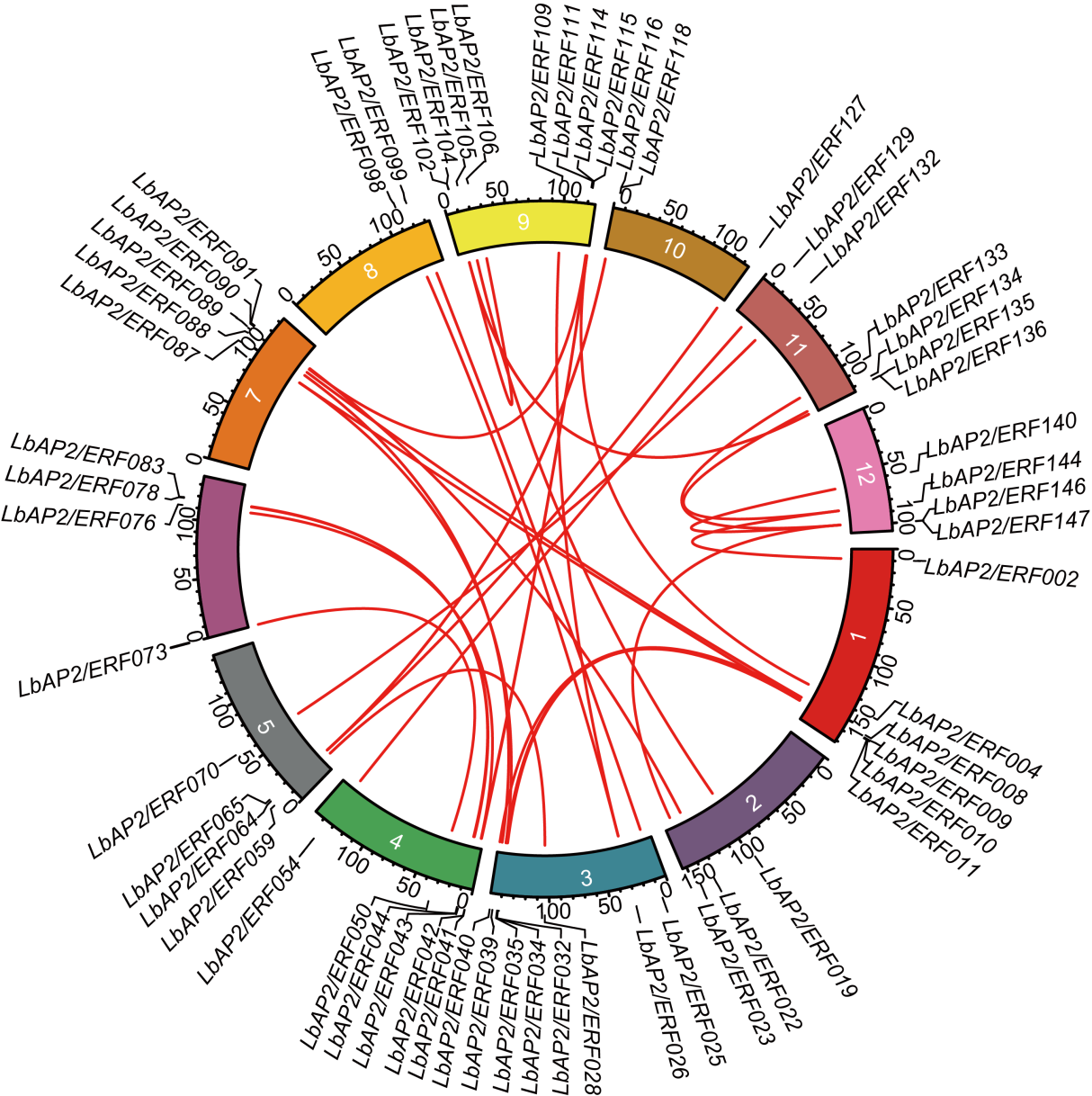


**Supplementary Figure 2.** Distribution of segmentally duplicated *LbAP2/ERF* genes on *L. barbarum* chromosomes. Red lines indicate duplicated *LbAP2/ERF* gene pairs.

**
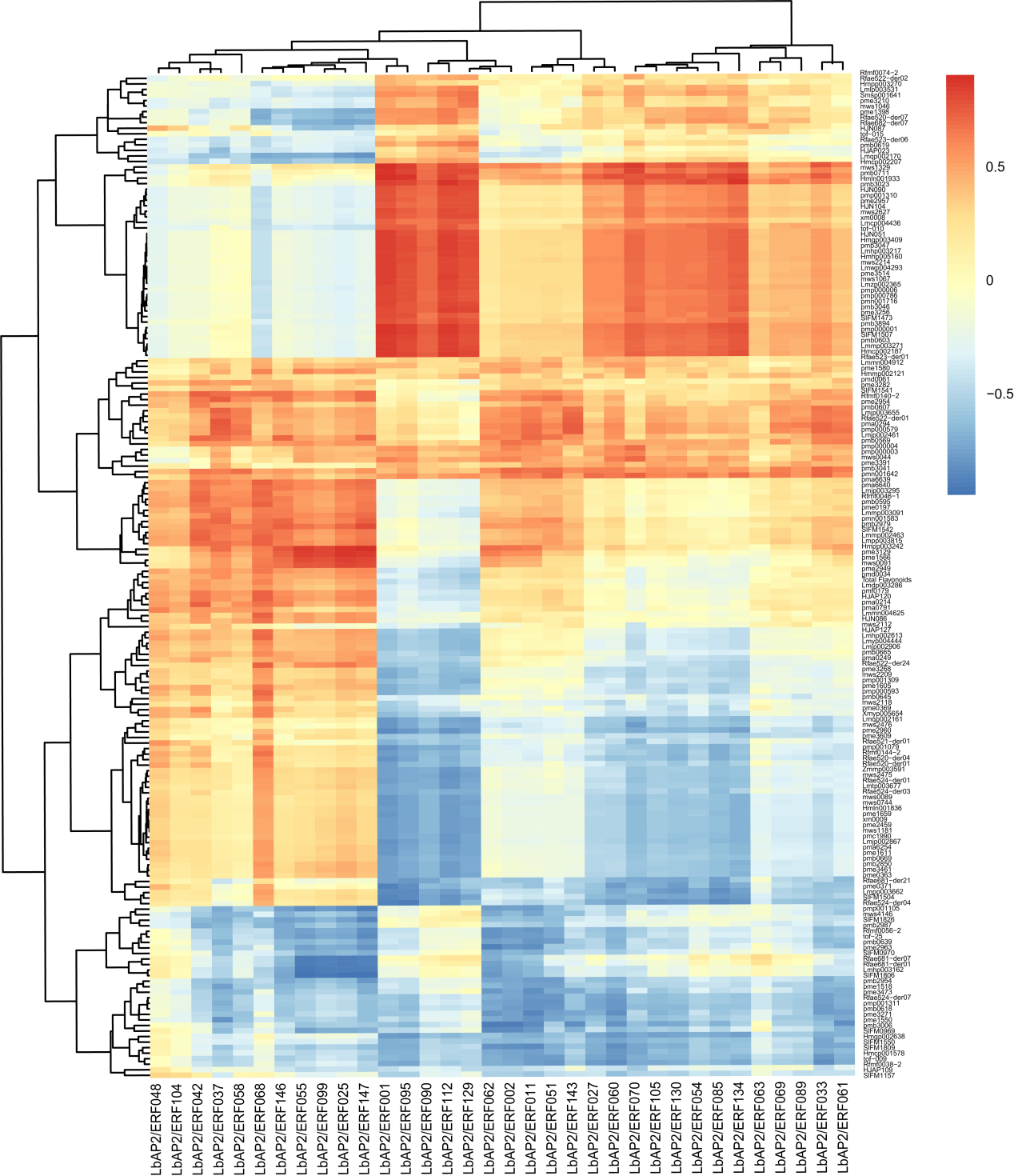
**

**Supplementary Figure 3.** Heatmap of the correlation between *LbAP2/ERF* genes and flavonoid metabolite accumulation. The color scale indicates correlation, from low (blue) to high (red).
